# Clinical characteristics and predictive factors of recurrent idiopathic transverse myelitis

**DOI:** 10.3389/fneur.2024.1416251

**Published:** 2024-09-19

**Authors:** Eun Kyoung Lee, Sooyoung Kim, Eunhee Sohn

**Affiliations:** ^1^Department of Neurology, Chungnam National University Sejong Hospital, Sejong, Republic of Korea; ^2^Department of Neurology, Chungnam National University Hospital, Daejeon, Republic of Korea

**Keywords:** idiopathic, transverse myelitis, recurrence, longitudinally extensive transverse myelitis, risk factor

## Abstract

**Background:**

Idiopathic transverse myelitis (iTM) is defined as an inflammatory myelopathy of undetermined etiology, even after a comprehensive workup to identify other possible causes. Generally, the characteristics of recurrent iTM are not clearly defined. This study aimed to identify the clinical characteristics and predictive factors of recurrence in patients with iTM.

**Methods:**

We retrospectively recruited patients with transverse myelitis (TM) who visited Chungnam National University Hospital between January 2011 and December 2021. We included patients who were followed up for at least 2 years and excluded those diagnosed with multiple sclerosis or neuromyelitis optica spectrum disorder (NMOSD) during the initial episode or follow-up period. Patients with iTM were categorized into two groups: monophasic idiopathic TM (mTM) and recurrent idiopathic TM (rTM). We compared the clinical characteristics and spinal magnetic resonance imaging findings between the two groups.

**Results:**

In total, 167 patients were reviewed, of whom 112 were excluded. Finally, we included 55 patients with iTM. In 55 patients, 11 (20.0%) and 44 (80%) were classified into the rTM and mTM groups, respectively. Male predominance was observed in the iTM, rTM, and mTM groups. The percentage of patients with low vitamin D levels was significantly higher in the rTM group (100.0%) compared with the mTM group (70%) (*p* = 0.049). In addition, longitudinally extensive transverse myelitis (LETM) was observed more frequently in the rTM group, in 8 of 11 (72.7%) patients, compared with 15 of 44 (34.1%) patients in the mTM group, with the difference being statistically significant (*p* = 0.020). In multivariate regression analysis, female sex, younger age at onset, low serum vitamin D level (<30 ng/mL), and LETM were risk factors for recurrence. LETM was a significant predictor of relapse in iTM (*p* = 0.043, odds ratio = 13.408).

**Conclusion:**

In this study, the clinical features of mTM and rTM are nearly indistinguishable. In conclusion, >20% of the patients with iTM experience recurrence, and LETM is the most significant risk factor for recurrence. In cases of recurrence, there is a favorable response to immunotherapy, and the prognosis is generally good. Although LETM may be the initial symptom of NMOSD, it may be manifestation of iTM, and in cases of idiopathic LETM, it is important to be mindful of the elevated risk of recurrence. Based on these results, idiopathic rTM has good clinical prognosis and response to immunosuppressive treatment.

## 1 Introduction

Transverse myelitis (TM) is an inflammatory disorder of the spinal cord that results from several etiologies, such as multiple sclerosis (MS), neuromyelitis optica spectrum disorder (NMOSD), infectious diseases, and autoimmune rheumatologic disorders (1). Idiopathic transverse myelitis (iTM) is defined as an inflammatory myelopathy of undetermined etiology, even after a comprehensive workup to identify other possible causes. Although iTM is generally considered a monophasic disease, recurrent iTM has also been reported (2). Predicting recurrence in patients with iTM for whom no specific causes have been identified is challenging. However, a second attack can cause severe disability in patients who already have neurological impairment with reduced quality of life. Therefore, it is necessary to continuously follow up and repeatedly examine patients with a high risk of recurrence.

According to previous studies on demographic features or biomarkers associated with TM recurrence, African-American race, female sex, longitudinally extensive transverse myelitis (LETM) at onset, anti-Ro (Sjögren’s-syndrome-related antigen A) antibodies, vitamin D insufficiency, antinuclear antibody (ANA) titer, and immunoglobulin G (IgG) index have been suggested as risk factors for recurrence. However, these studies have included patients who were finally diagnosed with NMOSD or MS (3, 4). To date, few studies have been published on the risk factors for the recurrence and predictive factors of iTM.

We aimed to identify the risk factors and predictive biomarkers for iTM recurrence. To ensure the specificity of iTM, we included only patients who were followed up for >2 years without identified causes. We compared the clinical features and magnetic resonance imaging (MRI) findings between recurrent and monophasic iTM.

## 2 Materials and methods

### 2.1 Patients and clinical data

**Figure 1:**
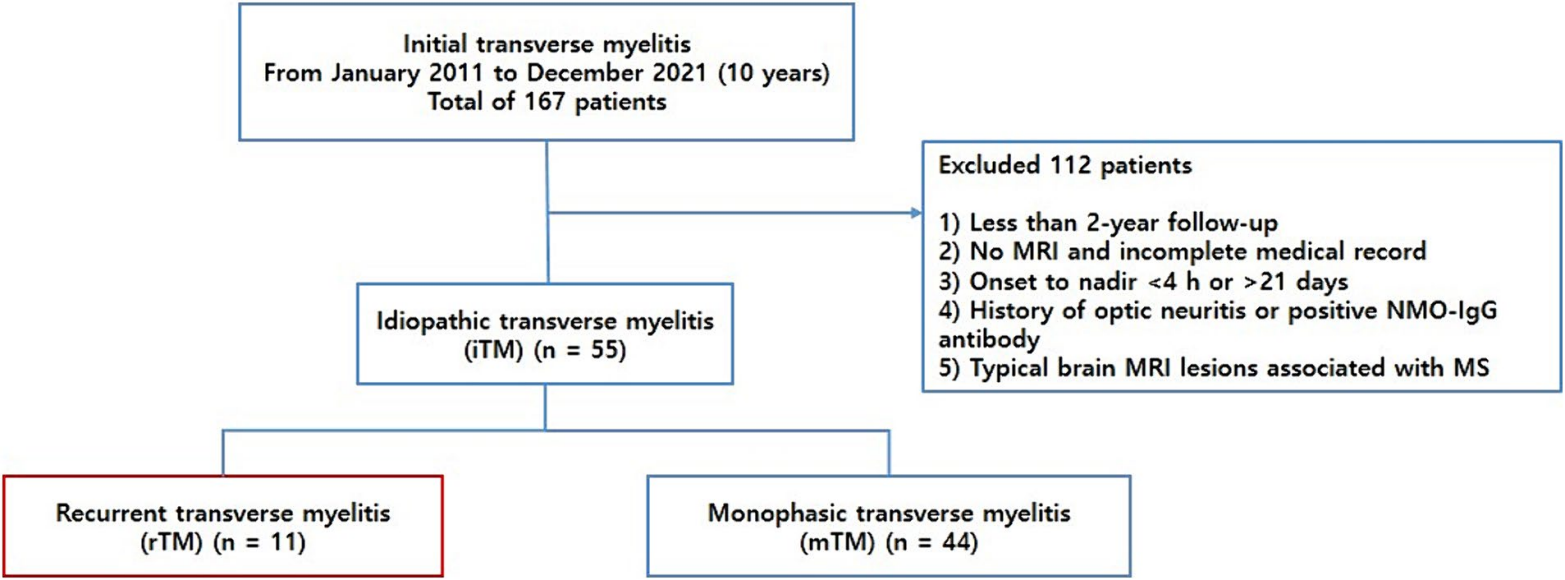
Flowchart of the patients included.

We retrospectively recruited patients with TM who visited Chungnam National University Hospital between January 2011 and December 2021. All the patients met the Transverse Myelitis Consortium Working Group diagnostic criteria for iTM (5). To meet the criteria of “acute myelitis,” only those who progressed to nadir between 4 h and 21 days following the onset of symptoms were included. All potential causes of acute myelopathy, including compressive, vascular, viral infections, metabolic, paraneoplastic, radiation, and vasculitis associated myelopathies, were excluded. To specifically focus on iTM, we included patients who were followed up for at least 2 years and excluded those diagnosed with MS or NMOSD during the initial episode or follow-up period. TM along with a history of optic neuritis or positive anti-aquaporin 4 (AQP4) IgG antibodies was classified as NMOSD and excluded from the study population. Patients with typical cerebral MRI lesions associated with MS were excluded from this study. Finally, the patients with iTM were categorized into two groups: monophasic idiopathic TM (mTM) and recurrent idiopathic TM (rTM) (Figure 1). Recurrence was defined as the development of sensory, motor, or autonomic dysfunction attributable to the spinal cord and new lesions demonstrated on spinal MRI with gadolinium enhancement.

All patients with iTM were treated with intravenous (IV) methylprednisolone during the acute stage. To prevent further relapse, patients diagnosed with recurrent iTM continued immunosuppressive treatments, such as azathioprine, mycophenolate mofetil, or oral prednisolone, after recurrence.

We collected various clinical and laboratory data, including sex, age at onset, duration of illness, clinical symptoms, Expanded Disability Status Scale (EDSS) scores, cerebrospinal fluid (CSF) oligoclonal band (OCB), IgG index, anti-AQP4 antibodies, anti-myelin oligodendrocyte glycoprotein (MOG) antibodies, CSF white blood cell count and protein levels, anti-Ro/La antibodies, and serum IgE and vitamin D levels. The follow-up period in our hospital from symptom onset was defined as the disease duration. The EDSS score was measured at the onset, nadir, and after treatment at the last visit (Table 1). Additionally, we reviewed the spinal cord and cerebral MR images of all patients.

**Table 1:** Clinical characteristics of the 55 patients with idiopathic transverse myelitis.

iTM (*N* = 55)	rTM (*n* = 11)	mTM (*n* = 44)	*p*-value
Male (%)	6/11 (54.5)	34/44 (77.3)	0.130
Onset age (years)	45.5 ± 14.0	52.1 ± 15.6	0.185
Disease duration (months)	76.7 ± 59.8	38.0 ± 24.9	0.053
Onset to nadir (months)	0.1 ± 0.3	0.4 ± 0.5	0.047^*^
Onset to recur (months)	16.0 ± 13.2	None	–
Initial presenting symptom (%)	–	–	0.425
Sensory involvement	8/11 (72.7)	27/44 (61.4)	
Motor involvement	0/11 (0.0)	3/44 (6.8)	
Sensory + motor	3/11 (27.3)	8/44 (18.2)	
Sensory + motor + autonomic	0/11 (0.0)	6/44 (13.6)	
Neuropathic pain (+)	10/11 (90.9)	30/44 (68.2)	0.130
EDSS at onset	1.5 ± 0.2	1.5 ± 0.4	0.424
EDSS at nadir	2.0 ± 0.2	1.9 ± 0.6	0.266
EDSS after treatment	1.4 ± 0.2	1.2 ± 0.5	0.088
CSF OCB (+) (%)	2/11 (18.2)	7/39 (17.9)	0.986
IgG index >0.7	2/9 (22.2)	9/40 (22.5)	0.986
Aquaporin 4 antibodies (+) (%)	0/8 (0.0)	0/39 (0.0)	–
Anti-MOG antibodies (+) (%)	1/6 (16.7)	1/16 (6.3)	0.636
CSF WBC >5	2/10 (20.0)	6/39 (15.4)	0.725
CSF protein (mg/dL)	28.3 ± 12.7	40.1 ± 15.2	0.016^*^
Anti-Ro antibodies (+) (%)	0/11 (0.0)	1/43 (2.3)	0.610
Anti-La antibodies (+) (%)	0/11 (0.0)	0/43 (0.0)	–
Serum IgE level (IU/mL)	323.2 ± 255.3	223.8 ± 231.3	0.187
Vitamin D level < 30 (ng/mL)	10/10 (100.0)	26/37 (70.3)	0.049*

Values are presented as means ± standard deviations (minimum–maximum) or numbers (%).

EDSS, Expanded Disability Status Scale; CSF, cerebrospinal fluid; OCB, oligoclonal band; anti-MOG, anti-myelin oligodendrocyte glycoprotein; WBC, white blood cell.

^*^ Statistically significant results (*p* < 0.05).

### 2.2 Magnetic resonance imaging (MRI) evaluation

MRI of the brain and spinal cord was performed at the first diagnosis and when recurrence was suspected. MR images were obtained using four 3 T MRI scanners with 32 channels: Discovery MR 750 W (GE Healthcare, Waukesha, WI, United States), MAGNETOM Skyra (Siemens Medical Solutions, Erlangen, Germany), Ingenia Elition X (Philips Healthcare, The Netherlands), and Achieva (Philips Healthcare). MRI of the spinal cord with T1-weighted imaging (T1WI) and contrast-enhanced T1WI, T2-weighted imaging (T2WI), and T2WI sagittal 3-mm cuts were evaluated. All images were independently analyzed by three neurologists (Lee EK, Kim S, and Sohn E). The number and location of the involved segments were evaluated for enhancement and swelling of the spinal cord using spinal MRI. The involvement of ≥3 vertebral segments on T2WIs was referred to as LETM. All cases with abnormal findings on the brain MRI were excluded.

### 2.3 Statistical analyses

All statistical analyses were performed using the IBM SPSS Statistics for Windows (version 26.0; IBM Corp., Armonk, N.Y., United States). Statistics for the clinical variables are presented as proportions, means, standard deviations, and ranges. The independent t-test, Pearson’s chi-squared test, Fisher’s exact test, Mann–Whitney U test, and correlation analysis were used to compare the statistical significance between the groups. Logistic regression analysis was performed to determine the predictive factors for rTM. We calculated odds ratios (ORs) and their respective 95% confidence intervals (CIs). Statistical significance was set at *p* < 0.05.

## 3 Results

### 3.1 Patient baseline characteristics

In total, 167 patients were reviewed, and 112 were excluded based on the provided exclusion criteria (Figure 1). We ultimately included 55 patients with iTM, with a mean age of 50.8 ± 15.4 years and a male:female sex ratio of 3.1:1. In 55 patients, 11 (20.0%) and 44 (80%) were classified into the rTM and mTM groups, respectively. A male predominance was observed in the iTM, rTM, and mTM groups (72.7, 54.5, and 77.3%, respectively). The mean onset ages of the rTM and mTM groups were 45.5 ± 14.0 and 52.1 ± 15.6 years, respectively. In the comparison of clinical characteristics between the rTM and mTM groups, the duration of onset to nadir of the rTM group was 0.1 ± 0.3 month, which was significantly shorter than of the mTM group, which was 0.4 ± 0.5 month (*p* = 0.047). However, there were no significant differences in follow-up duration, initial presenting symptoms, presence of neuropathic pain, and EDSS scores between the two groups.

In the analysis of laboratory findings, CSF OCB positivity, IgG index, and serum IgE levels were not significantly different between the two groups. All patients were AQP4 antibody-negative, and one patient in each of the two groups tested positive for MOG antibodies. The CSF protein level was lower in the rTM group than in the mTM group; however, the increase was not clinically significant. One patient had anti-Ro antibodies but was not diagnosed with Sjögren’s disease because he did not reveal other clinical features of Sjögren’s disease. The percentage of patients with low vitamin D levels was significantly higher in the rTM group (100.0%) compared with the mTM group (70%) (*p* = 0.049) (Table 1).

### 3.2 Spinal MRI lesion analysis

The involved cord segment counts were 3.1 ± 1.5 in the rTM group and 2.2 ± 1.6 in the mTM group, with statistical significance (*p* = 0.044) (Table 2). In addition, LETM was observed more frequently in the rTM group (8 of 11 [72.7%] patients) than in the mTM group (15 of 44 [34.1%] patients), and this difference was statistically significant (*p* = 0.020) (Table 2). However, the location of the involved segment, enhancement, and swelling of the lesion were not significantly different between the two groups.

**Table 2:** Comparison of spinal MRI findings between the rTM and mTM groups.

iTM (*N* = 55)	rTM (*n* = 11)	mTM (*n* = 44)	*p*-value
Number of involved cord segment	3.1 ± 1.5	2.2 ± 1.6	0.044^*^
LETM (%)	8/11 (72.7)	15/44 (34.1)	0.020^*^
Involved segment	–	–	0.562
Cervical involvement	6/11 (54.5)	20/44 (45.5)	–
Thoracic involvement	5/11 (45.5)	20/44 (45.5)	–
Cervical and thoracic involvement	0/11 (0.0)	4/44 (9.0)	–
Location	–	–	0.100
Partial cord involvement	3/11 (27.3)	19/44 (43.2)	–
Central cord involvement	1/11 (9.1)	12/44 (27.3)	–
Total involvement	7/11 (63.6)	13/44 (29.5)	–
Enhancement	10/10 (100.0)	34/40 (85.0)	0.192
Swelling	9/11 (81.8)	23/44 (52.3)	0.076

Values are presented as means ± standard deviations (minimum–maximum) or numbers (%).

LETM, longitudinally extensive transverse myelitis; mTM, monophasic idiopathic transverse myelitis; rTM, recurrent idiopathic transverse myelitis.

^*^Statistically significant results (*p* < 0.05).

### 3.3 Multivariate regression analysis

Logistic regression analysis was performed to identify risk factors that significantly increased the recurrence of iTM (Table 3). In multivariate regression analysis, younger age at onset, female sex, rapid progression, and LETM were statistically significant risk factors for recurrence. Among these factors, LETM was an overall significant predictor of relapse in iTM (*p* value = 0.043, OR = 13.408) (Table 3).

**Table 3:** Multivariate logistic regression analysis predicting the recurrence of idiopathic transverse myelitis.

Recurrent TM	OR	95% CI	*p*-value
Male sex (%)	0.036	0.001–0.895	0.043^*^
Onset age (years)	0.866	0.757–0.991	0.036^*^
Onset to nadir (months)	0.049	0.002–1.137	0.060
LETM	13.408	1.087–165.396	0.043^*^
Vitamin D level < 30 (ng/dL)	0.000	0.000–0.000	0.999

LETM, longitudinally extensive transverse myelitis; OR, odds ratio; CI, confidence interval.

^*^ Statistically significant results (*p* < 0.05).

### 3.4 Clinical features and laboratory findings of 11 patients with recurrent idiopathic transverse myelitis

Among the 11 patients with rTM, three and eight had short-segment transverse myelitis (SSTM) and LETM, respectively (Table 4). All patients were treated with IV methylprednisolone (1 g/day) for 5 days with no further medication in the acute stage. Ten patients experienced a single recurrence, and one patient experienced three recurrences. After recurrence, they were treated with immunosuppressive medications. Ten of the 11 patients had low vitamin D levels, except for one patient who did not undergo testing (Table 4).

**Table 4:** Clinical features and laboratory findings of 11 patients with recurrent transverse myelitis (rTM).

Pt n.	Type	Sex	Age	Disease duration (months)	EDSS at nadir	N of recurrence	Symptom	Relapse prevention therapy	Maintenance treatment after recurrence	CSF OCB	IgG index	MOG Ab	Serum IgE U/mL	Serum vitamin D ng/mL
1	SSTM	M	36	104	2.0	1	Sensory	−	AZA	−	0.57	+	323.2	25.59
2	SSTM	F	40	33	1.5	1	Sensory	−	Steroid	+	0.43	−	292.7	12.13
3	SSTM	F	50	174	2.0	1	Sensory	−	Interferon ß - > (−)	−	None	None	None	None
4	LETM	F	73	80	2.5	1	Sensory + motor	−	Steroid + MMF	+	0.78	−	347.7	17.07
5	LETM	M	58	44	2.0	1	Sensory	−	Steroid	−	0.6	None	None	25.85
6	LETM	M	41	24	2.0	1	Sensory + motor	−	Steroid + AZA	−	0.93	None	280.5	21.6
7	LETM	M	48	49	2.0	3	Sensory + motor	−	Steroid + MMF	−	0.4	−	614.9	9.37
8	LETM	M	27	27	2.0	1	Sensory	−	AZA	−	0.61	None	815.0	21.02
9	LETM	F	43	24	2.0	1	Sensory	−	Steroid	−	0.4	−	71.33	23.96
10	LETM	F	58	92	2.0	1	Sensory	−	Steroid	−	0.61	−	7.77	13.85
11	LETM	M	26	193	2.0	1	Sensory	−	Interferon ß - > (−)	−	None	None	155.8	27.2

Pt, patient; *n*, number; SSTM, short-segment transverse myelitis; LETM, longitudinally extensive transverse myelitis; M, male; F, female; EDSS, Expanded Disability Status Scale; AZA, azathioprine; MMF, mycophenolate; (−), stop medication; CSF, cerebrospinal fluid; OCB, oligoclonal band; MOG, myelin oligodendrocyte glycoprotein; Ab, antibody; None, no test result.

## 4 Discussion

In this study, among the 55 patients who were diagnosed with iTM after >2 years of follow-up, 11 (20.0%) had recurrent myelitis. The clinical features of mTM and rTM were almost indistinguishable. However, rTM was associated with a younger age of onset, a higher proportion of females, a more rapid progression, a greater percentage of patients with low vitamin D levels, and more instances of LETM on MRI compared with mTM. Furthermore, regression analysis showed that LETM was the most significant risk factor for recurrence. In the rTM group, only one of 55 (1.8%) patients experienced recurrence three times, and the other 10 patients experienced one relapse. All of them responded well to immunosuppressive therapies and showed good EDSS score (1.4 ± 0.2) at final visit. Based on these results, idiopathic rTM can be considered to have good clinical prognosis and response to immunosuppressive treatment.

Idiopathic ATM was initially regarded as monophasic. However, it has recently reported that 25% of patients with ATM experience recurrence (6). Relapsing transverse myelitis usually occurs in MS, NMOSD, and other conditions, including systemic lupus erythematosus, herpes simplex infection, antiphospholipid antibody syndrome, and spinal arteriovenous malformation (6). Recent reviews have shown that MOG antibody disease-associated TM is also a cause of recurrent TM (7). However, to the best of our knowledge, no previous studies have focused on patients with recurrent iTM with no specific cause of TM. Kimbrough et al. (4) reported that 110 out of 192 (57%) patients with idiopathic TM developed recurrent symptoms. However, they enrolled patients initially diagnosed with iTM at onset, and the final diagnoses varied as follows: iTM (*n* = 34), NMOSD (*n* = 69), and systemic autoimmune disease (*n* = 7). Among the patients with relapse, only 34 of 192 (17.7%) were eventually diagnosed with recurrent iTM, which is consistent with the frequency of rTM (20.0%) in this study. They identified the following risk factors for recurrence: African-American race, female sex, LETM at onset, anti-Ro antibody positivity, vitamin D insufficiency, ANA, and presence of inflammatory markers (IgG index) in the CSF. These risk factors may be partly driven by the greater likelihood of developing NMOSD. In a study on the risk factors for recurrence in inflammatory myelitis, Marrodan et al. (8) reported that LETM increased risk of NMOSD and recurrence of idiopathic TM. However, they also included patients with NMOSD and MS in their analysis. In another study focusing on iTM, recurrent iTM was compared with MS-associated recurrent TM (9). The study found a recurrence rate of 40%, and recurrent iTM differed from MS-associated recurrent TM in terms of male predominance, absence of OCB, frequent multiple relapses, and frequent presentation of acute transverse myelitis. However, this study was conducted before the discovery of AQP4 antibodies, and NMOSD was not excluded from the study population. Therefore, predicting the extent to which patients with NMOSD may be included among those classified as having recurrent iTM is challenging. We observed a male predominance in our population, which is consistent with previous studies on TM among Koreans.

We identified LETM as the most significant risk factor for iTM recurrence. To date, few studies have specifically addressed idiopathic LTEM with negative AQP4 antibodies (10–13). The reported recurrence rate of idiopathic LETM with negative AQP4 expression ranges from 24.5 to 71% (6), which is comparable to or higher than the known recurrence rate of iTM. This finding suggests that LETM may be a risk factor for iTM recurrence. The clinical features of our patients with recurrent iTM, such as the absence of female predominance, less frequent relapse, benign clinical course, absence of combined autoimmunity, and relatively good response to immunosuppressive therapy, were consistent with those of previous studies (9, 11, 13, 14). These features are characteristics of idiopathic LETM compared with AQP4-positive LETM. Therefore, idiopathic LETM should be considered independent of AQP4-positive LETM, and patients presenting with LETM, even if AQP4 is negative, may still be at risk of recurrence.

Additionally, we observed that rTM resulted in a higher percentage of patients with low vitamin D levels compared with mTM. However, these factors did not reach statistical significance after the regression analysis. Mealy et al. reported an association between lower total vitamin D levels in patients with recurrent inflammatory spinal cord disease compared with those with monophasic disease (15). The possible immunomodulatory effects of vitamin D on NMOSD have also been postulated in a recent study (16). Further studies with larger patient populations will help elucidate the relationship between vitamin D level and iTM recurrence.

Recently, some studies have suggested that relapsing pure short-segment partial myelitis has a possibility of pure spinal MS (17, 18). A previous study suggested the following criteria for pure spinal MS: (1) relapsing short-segment partial myelitis, (2) positive CSF OCB, (3) MRI of the brain not meeting the 2017 McDonald criteria, (4) good response to MS disease-modifying therapies, and (5) all other alternative diagnosis ruled out (17). In this study, one patient with recurrent SSTM met the criteria for pure spinal MS. However, she did not experience recurrence after low-dose oral steroid therapy. Pure spinal MS has not been fully validated, and further studies are required.

The pathological mechanisms underlying the recurrence of iTM remain unclear. TM is an autoimmune-mediated swelling of myelin sheaths, directed by anti-myelin basic protein antibodies, anti-AQP-4, and anti-MOG antibodies (2). The reason for restricted pathological involvement of the spinal cord, except for the brainstem or cerebral parenchyma, in our patients remains unclear. These regions are not known to have different glial cells or myelin proteins compared with other parts of the CNS; however, regional differences in the permeability of the blood–brain barrier or the ability of antigen processing and presentation to activate T cells may explain this unique topography of involvement (19, 20).

This study has some limitations. First, as this was a retrospective study in a single center, the number of adult patients was small, and these patients were recruited from one Asian country. Therefore, racial and age differences were not considered in this study. Second, there is a possibility of underdiagnosis due to the relatively short follow-up period, with an average of 45.7 (range, 24–193) months in the study population. Another limitation is that MOG antibody testing, which has been identified as the cause of recurrent TM, could not be conducted in all patients. In this study, the MOG antibody test was performed in 22 of 55 (40%) patients. Because testing for MOG antibodies has become available only in recent years, patients who developed the disease many years earlier are unlikely to be diagnosed. However, in a study investigating the significance of MOG antibodies in patients with AQP4-negative LETM, the risk of optic neuritis relapse was higher in MOG-positive patients compared with MOG-negative patients; however, there were no significant differences in the recurrence of TM among the patients (21). Therefore, it has been hypothesized that MOG antibodies in AQP4-negative LETM may have a limited association with TM recurrence.

In conclusion, >20% of the patients with iTM experience recurrence, and LETM is the most significant risk factor for recurrence. In cases of recurrence, there is a favorable response to immunotherapy, and the prognosis is generally good. Although LETM may be the initial symptom of NMOSD, it may be distinct from NMOSD, and in cases of idiopathic LETM, it is important to be mindful of the elevated risk of recurrence. Further studies are required to understand the disease entity of recurrent TM and develop proper treatment strategies in the early stages of rTM.

## Data Availability

The original contributions presented in the study are included in the article/supplementary material, further inquiries can be directed to the corresponding author.
